# Substrate Specificity and Enzyme Recycling Using Chitosan Immobilized Laccase

**DOI:** 10.3390/molecules191016794

**Published:** 2014-10-17

**Authors:** Everton Skoronski, Mylena Fernandes, Maria de Lourdes Borba Magalhães, Gustavo Felippe da Silva, Jair Juarez João, Carlos Henrique Lemos Soares, Agenor Fúrigo Júnior

**Affiliations:** 1 Laboratório de Tratamento de Águas e Efluentes, Departamento de Engenharia Ambiental, Universidade do Estado de Santa Catarina, Lages 88520-000, Brazil; 2 Laboratório de Engenharia Bioquímica, Departamento de Engenharia Química e Engenharia de Alimentos, Universidade Federal de Santa Catarina, Florianópolis 88040-900, Brazil; E-Mails: mylena.fernandes@gmail.com (M.F.); agenor@enq.ufsc.br (A.F.J.); 3 Laboratório de Bioquímica, Departamento de Produção Animal e Alimentos, Universidade do Estado de Santa Catarina, Lages 88520-000, Brazil; E-Mails: maria.borba.magalhaes@gmail.com (M.L.B.M.); gustavo.silva@udesc.br (G.F.S.); 4 Grupo de Catálise Enzimática e Síntese Orgânica, Departamento de Engenharia Química, Universidade do Sul de Santa Catarina, Tubarão 88704-900, Brazil; E-Mail: jair.joao@unisul.br; 5 Laboratório de Avaliação Exotoxicológica, Departamento de Bioquímica, Universidade Federal de Santa Catarina, Florianópolis 88040-900, Brazil; E-Mail: chsoares@ccb.ufsc.br

**Keywords:** laccase, *Aspergillus* sp., chitosan, enzyme immobilization, phenolic compounds, bioremediation

## Abstract

The immobilization of laccase (*Aspergillus* sp.) on chitosan by cross-linking and its application in bioconversion of phenolic compounds in batch reactors were studied. Investigation was performed using laccase immobilized via chemical cross-linking due to the higher enzymatic operational stability of this method as compared to immobilization via physical adsorption. To assess the influence of different substrate functional groups on the enzyme’s catalytic efficiency, substrate specificity was investigated using chitosan-immobilized laccase and eighteen different phenol derivatives. It was observed that 4-nitrophenol was not oxidized, while 2,5-xylenol, 2,6-xylenol, 2,3,5-trimethylphenol, syringaldazine, 2,6-dimetoxyphenol and ethylphenol showed reaction yields up 90% at 40 °C. The kinetic of process, enzyme recyclability and operational stability were studied. In batch reactors, it was not possible to reuse the enzyme when it was applied to syringaldazne bioconversion. However, when the enzyme was applied to bioconversion of 2,6-DMP, the activity was stable for eight reaction batches.

## 1. Introduction

Assessment and treatment of environmental phenol contamination is of great importance, since phenols are highly toxic compounds for most living organisms and considered persistent and bio accumulative in the environment. Phenolic compounds are found in wastewater of many industries such as the textile, paper, petrochemical, plastics, resins, pharmaceutical and pesticide industries, where typical concentrations range between 100 and 1000 mg·L^−1^ [1].

Due to their high toxicity, bioremediation of phenolic compounds is usually hampered when environmental concentrations rise above 100 mg·L^−1^. In such cases, physico-chemical methods represent a remarkable alternative for phenol removal from contaminated effluents [2]. However, such decontamination strategies are usually expensive and therefore, the improvement and development of new strategies for phenol remediation in the environment is crucial.

The use of enzymatic strategies whereby phenolic compounds are oxidized by peroxidases or polyphenoloxidases has shown promising results [3,4,5,6]. Polyphenoloxidases catalyze hydroxylation of monophenols producing the respective *o*-diphenol, which is further converted into an *o*-quinone in the presence of oxygen. These compounds are further polymerized and precipitated, being easily removed from solution by flocculation [7].

Laccases (EC 1.10.3.2) are polyphenol oxidases that oxidize many organic and inorganic substrates including monophenols, polyphenols, amines, methoxyphenols and aromatic amines. This class of enzymes utilizes molecular oxygen as the final electron acceptor, culminating with production of a water molecule. The use of polyphenoloxidases appears as a more attractive strategy as opposed to the use of peroxidases, since in the latter case, hydrogen peroxide is used as the oxidizing agent [8].

The use of laccases in wastewater phenol remediation has been investigated [9] and has shown great promise for the treatment of textile [10,11], paper [12], leather [13] and oil industrial effluents [14,15]. The use of laccase for effluent treatment is indeed advantageous since allows the removal of recalcitrant chemicals from the environment such as aromatic xenobiotics, which are usually difficult to degrade. Additionally, laccases catalyze decoloration of clorophenol and textile industrial dyes, catalyze aromatic ring cleavage, and promote polycyclic aromatic hydrocarbon (PAH) mineralization [9] as well as phenol polymerization forming insoluble aggregates that can be easily removed by flocculation or filtration [16].

Due to the high chemical complexity of industrial effluents, phenolic compounds are often subjected to several *in situ* chemical modifications producing different phenolic substituents and therefore, a detailed investigation of laccase substrate specificity towards different substituents is of great importance for the field [17,18,19,20].

Although studies have investigated the use of laccase in remediation of environmental phenols, we still lack detailed information about the influence of different substrate functional groups on laccase enzymatic activity, particularly when enzymes are covalently immobilized on solid supports. In fact, most studies of phenol oxidation have been performed using free enzymes, and our current knowledge of phenol oxidation using immobilized enzymes is still is very limited [21].

The use of immobilized enzymes is advantageous as compared to the use of free enzymes since allows easy enzyme recycling at the end of the process, improves stability and enzymatic robustness, enables continuous production and assures the absence of the biocatalyst in the product stream. Although several enzyme immobilization strategies have already been developed including chemical cross-linking, physical adsorption and polymeric encapsulation; the use of chemical crosslinking provides important advantages since provides additional stability against pH and ionic fluctuations and the enzyme remains covalently attached to the solid support, avoiding molecular diffusion and the consequent losses during sequential enzymatic cycles [22,23,24,25]. However, a decrease in enzymatic activity can be observed, since chemical crosslinking may cause deleterious structural changes on the enzyme tridimensional structure. Recent studies demonstrated that laccase can be immobilized into silica supports retaining more than 90% of enzymatic activity as compared to the soluble enzyme [26]. Such porous materials are widely used and can be utilized for enzyme immobilization via silanization using their surface exposed OH groups [27]. However, these solid supports present limitations regarding their industrial applications due to elevated production costs and low stability in continuous flow systems [28]. Therefore, in this work chitosan immobilized laccase was investigated and the enzymatic performance of immobilized laccase using different phenol derivatives was examined.

## 2. Results and Discussion

### 2.1. Enzymatic Oxidation of Phenolic Compounds Using Immobilized Laccase

The presence of organic contaminants in wastewater effluents has become a matter of growing concern because of their potential deleterious effects on human health and environment. In general, industrial effluents are not simple mixtures, but instead, comprise a large number of phenolic wastewater contaminants and remediation of such complex mixtures may require elaborated strategies. Although investigation of laccase substrate specificity to predict effective contaminant removal from wastewaters is not a straightforward strategy, such information will certainly contribute to a better assessment of laccase-mediated decontamination. However, as important as determining laccase spectrum of action is in fact the molecular dissection of individual components from the complex mixtures of effluents. Interestingly, recent work has shown that wastewaters compounds may act as redox mediators further improving conversion of compounds recalcitrant to laccase oxidation [29].

Several studies have been performed utilizing free laccase to oxidize phenol derivatives [29,30,31,32]. In the present study, we have investigated *how* different substituents on the *aromatic ring* can *influence* the enzymatic activity of laccase immobilized on the solid support chitosan. Laccase was immobilized via chemical crosslinking due to higher enzymatic operational stability of this method as compared to immobilization via physical adsorption. Unspecific adsorption of phenolic compounds into crosslinked beads were observed and ranged from 0% to 1.2% among the various compounds and unspecific absorption was considered when activity was calculated. The enzymatic measurements utilized in this work rely on the estimation of substrate consumption by measurement of soluble phenol compounds after laccase mediated oxidation which leads mostly to the formation of insoluble phenol products not detected by our assays. However, this might lead to an underestimation of substrate consumption as soluble phenol derivatives might also be produced as previously reported [24]. Therefore, the use of HPLC based detection methods would be advantageous in order to avoid underestimation of substrate consumption in laccase mediated phenol oxidation reactions.

Obtained data demonstrate that the functional groups are crucial for enzyme activity using free as well as immobilized laccase (Table 1). Among tested substrates, 2,3-xylenol, 2,5-xylenol, 2,6-xylenol, 2,3,5-trimethylphenol and *p*-ethylphenol presented higher conversion rates using free as well as immobilized laccase. Conversely, some substrates presented very low conversion rates as in the case of phenol that presented only 7% and 31% conversion using immobilized and free enzyme, respectively. In the case of 4-nitrophenol, free laccase presented 12% conversion, while no conversion was observed using the immobilized enzyme. Although substrate specificity has not changed significantly upon enzyme immobilization, comparison of enzymatic rates using free *versus* immobilized laccase demonstrates that the overall conversion rates are diminished upon immobilization, which can be explained by decreased enzyme mobility and reduced intermolecular collisions. In fact, laccase catalytic efficiency (k_cat_/K*_m_*) using syringaldazine as substrate was 158 M^−1^·s^−1^ for the immobilized enzyme and 7830 M^−1^·s^−1^ using the free enzyme.

**Table 1:** Obtained results for enzymatic conversion of phenolic derivatives using immobilized and free laccase. Obtained data correspond to the percentage of substrate consumption at different temperatures where each data represent the average of three independent experiments. The respective standard errors are indicated. Reaction time: 24 h.

Phenolic Compound	Molecular Weight(g·mol^−1^)	% Conversion (Immobilized Enzyme)			% Conversion (Free Enzyme)		
		20 °C	30 °C	40 °C	20 °C	30 °C	40 °C
*o*-Cresol	108	12 ± 2	18 ± 5	45 ± 3	46 ± 3	46 ± 9	62 ± 2
*m*-Cresol		15 ± 5	14 ± 2	17 ± 2	27 ± 1	32 ± 10	44 ± 7
*p*-Cresol		5 ± 3	43 ± 10	24 ± 10	18 ± 4	72 ± 9	48 ± 5
*o*-Ethylphenol	122	15 ± 8	34 ± 7	7 ± 1	55 ± 1	78 ± 7	32 ± 3
*p*-Ethylphenol		39 ± 7	51 ± 3	100 ± 0	85 ± 7	92 ± 8	100 ± 0
2-Isopropylphenol	136	29 ± 4	35 ± 7	64 ± 10	78 ± 9	81 ± 2	92 ± 2
2,3,5-Trimethylphenol		82 ± 4	89 ± 1	90 ± 9	100 ± 0	100 ± 0	100 ± 0
4-Hydroxybenzoic acid	138	27 ± 9	32 ± 5	35 ± 6	40 ± 1	62 ± 1	80 ± 1
4-Nitrophenol	139	n.d.	n.d.	n.d.	5 ± 1	8 ± 4	12 ± 3
2,3-Xylenol	122	22 ± 10	47 ± 6	83 ± 1	55 ± 6	89 ± 2	100 ± 0
2,4-Xylenol		25 ± 3	49 ± 5	65 ± 1	32 ± 7	55 ± 3	81 ± 10
2,5-Xylenol		40 ± 1	70 ± 1	94 ± 3	52 ± 00	85 ± 4	100 ± 0
2,6-Xylenol		65 ± 8	92 ± 3	97 ± 6	91 ± 7	100 ± 0	100 ± 0
3,4-Xylenol		70 ± 7	85 ± 2	42 ± 10	52 ± 6	68 ± 5	27 ± 3
3,5-Xylenol		16 ± 3	27 ± 9	30 ± 7	28 ± 5	32 ± 9	55 ± 3
Phenol	94	5 ± 1	5 ± 2	7 ± 2	20 ± 3	26 ± 1	31 ± 7
Syringaldazine	360	100 ± 0	100 ± 0	100 ± 0	100 ± 0	100 ± 0	100 ± 0
2,6-DMP	154.16	100 ± 0	100 ± 0	100 ± 0	100 ± 0	100 ± 0	100 ± 0

**Figure 1:**
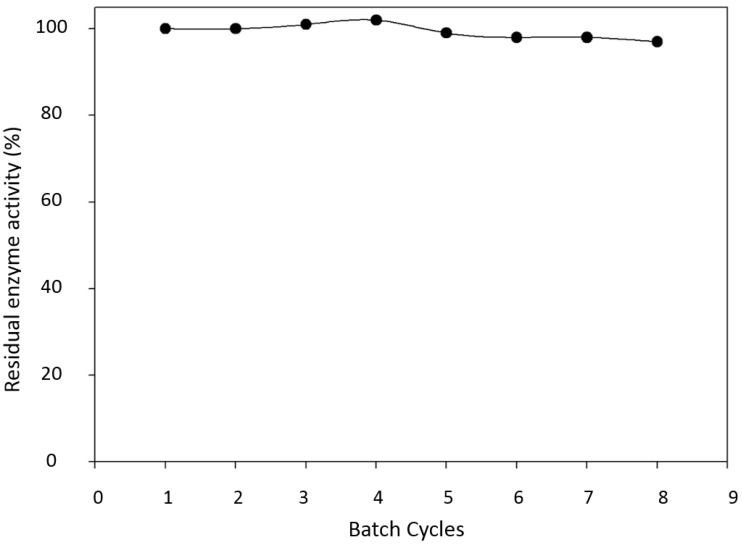
Residual enzyme activity after batch cycles of 2,6-DMP oxidation. Temperature of 40 °C, substrate concentration of 62.5 µmol·L^−1^ and enzyme concentration of 4.8 U·mL^−1^.

**Figure 2:**
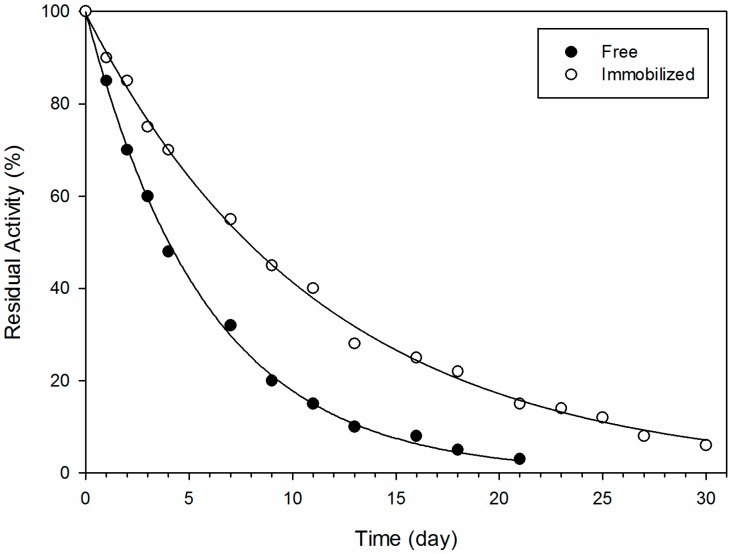
Residual enzyme activity after storage at 8 °C. Reactions were performed using syringaldazine as substrate, at room temperature and enzyme concentration of 4.8 U·mL^−1^.

Although conversion rates are indeed decreased and catalytic efficiency presents a fifty fold decrease for immobilized *versus* free enzyme, the observed catalytic losses are less significant when compared to the benefits created from the high enzymatic operational stability which allowed the recycling of the enzyme during eight enzymatic cycles without any loss of enzymatic activity (Figure 1). Furthermore, enzymatic immobilization leads to improved enzymatic stability over a period of 30 days as compared to the free enzyme (Figure 2).

Substrate specificity results are in accordance with previous data described by Felici and coworkers [33] and Xu [31] that have investigated substrate specificity of laccase from *Agaricus bisporus*. In the early report [33], authors have revealed that 4-nitrophenol is a poor substrate for laccase mediated oxidation, since only 2% substrate consumption was detected after 30 min incubation. Likewise, phenol was only 10% consumed after 30 min incubation at pH 6.0. In the later study [31] the authors have correlated laccase substrate specificity with the respective substrate redox potential. It was demonstrated that the rate limiting step of the enzymatic reaction is in fact the transference of the first electron from phenol to the cupper atom located at the enzyme active site [31]. Therefore, different ring substituents will provide different redox potentials to the phenolic compounds, which will in turn change the respective enzymatic efficiency towards each substrate. For example, in the case of 4-nitrophenol, the presence of an electron withdrawing group will decrease electron density located at the phenoxy group, which will hamper the substrate oxidation due to diminished electron availability.

Although 4-hydroxybenzoic acid also bears an electron withdrawing group, this compound is more acidic (pKa = 4.48) as compared to 4-nitrophenol (pKa = 7.2) [34], which may explain better conversion rates of about 30%. Furthermore, the position of ring substituent directly influences the enzyme efficiency. In the case of *m*-cresol, conversion rates no greater than 17% were observed, while its isomers *o*-cresol and *p*-cresol yielded conversion rates of 45% (40 °C) and 44% (30 °C), respectively (Table 1). This effect is also observed when compounds *o*-ethylphenol and *p*-ethylphenol were tested. Interestingly, in both cases symmetric molecules tend to produce better substrates for enzymatic oxidation.

### 2.2. Kinetic Studies

**Figure 3:**
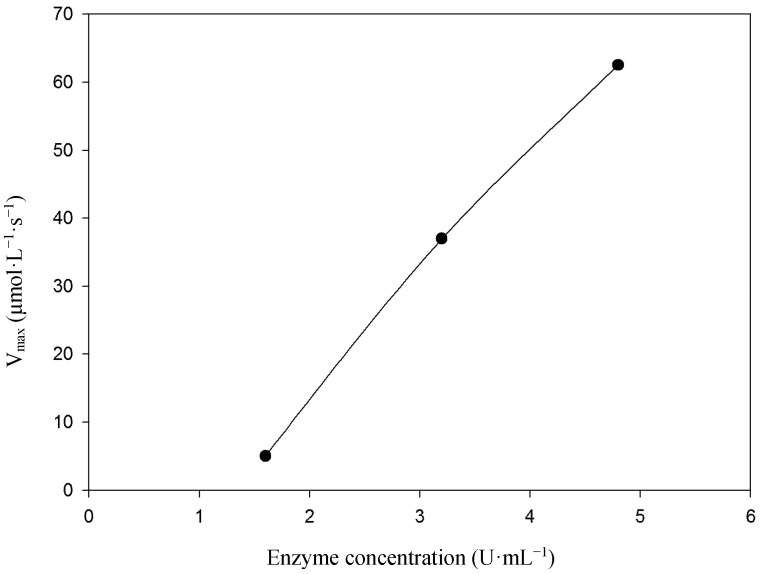
The influence of enzyme concentration on the velocity rate of syringaldazine oxidation. Temperature of 40 °C.

**Figure 4:**
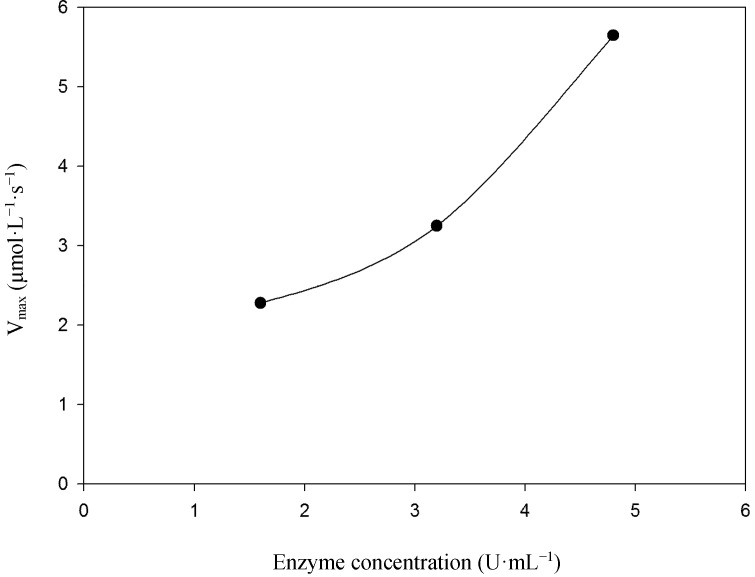
The influence of enzyme concentration on the velocity rate of 2,6-DMP oxidation. Temperature of 40 °C.

**Figure 5:**
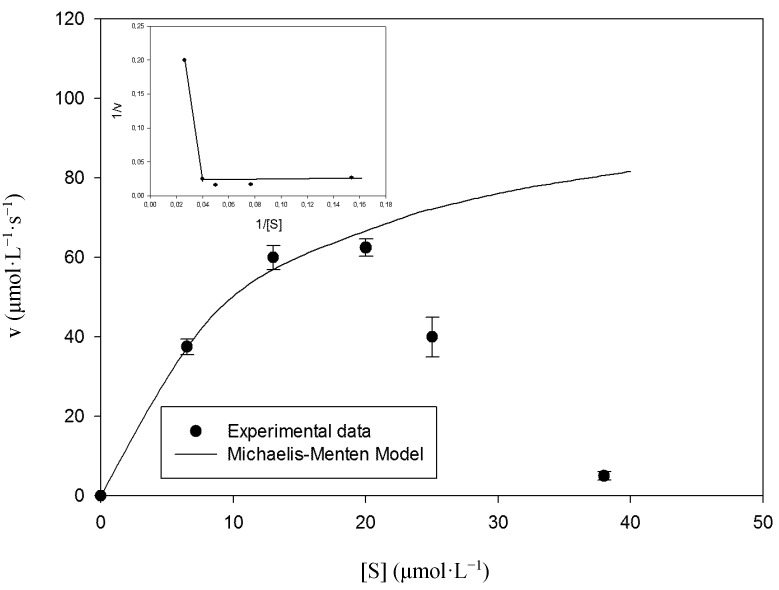
The influence of syringaldazine concentration on the initial reaction rates. Temperature of 40 °C and enzyme concentration of 4.8 U·mL^−1^. Each symbol (●) represents the average of three experimentally determined initial velocity measurements while error bars represent the respective standard deviation parameters. The line represents the fit of the data to Equation (2). As can be observed the enzyme does not present a linear behavior by double reciprocal analysis, indicating substrate inhibition (inset).

A plot of steady-state velocity *vs.* enzyme concentration was linear over the used concentration range using syringaldazine (Figure 3) as well as 2,6-DMP (Figure 4) as substrates, demonstrating velocity rates increase as the enzyme concentration rises. Analysis of substrate saturation curve using syringaldazine demonstrates the occurrence of enzyme inactivation at higher substrate concentrations (Figure 5).

This behavior was observed for all tested temperatures and is a common phenomenon observed in enzymology. Although syringaldazine has been used as a standard substrate for laccase enzymatic activity since 1973 [35], this is the first time substrate inhibition behavior has been described for the immobilized enzyme.

**Figure 6:**
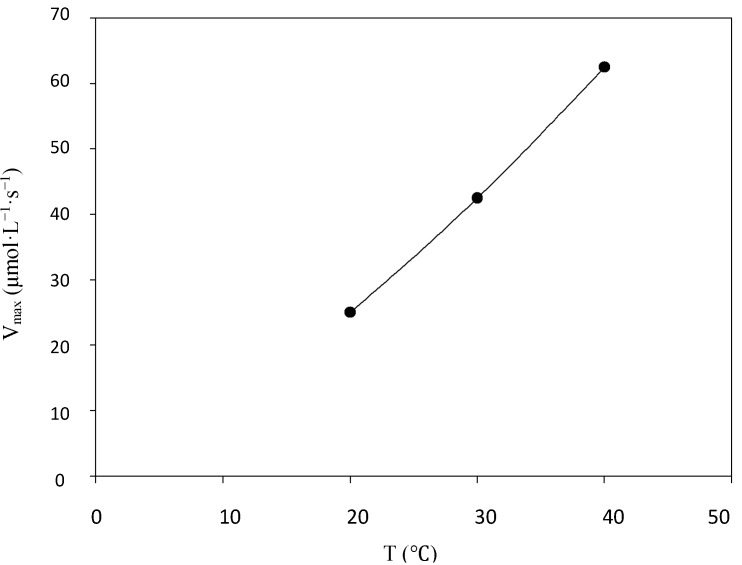
The influence of temperature on the velocity rate of syringaldazine oxidation. Enzyme concentration of 4.8 U·mL^−1^.

Temperature dependence on enzymatic activity was investigated (Figure 6). *v_max_* increases as the temperature rises from 20, 30 and 40 °C yielding of *v_max_* 25.0, 42. 5 and 62.5 µmol·L^−1^·s^−1^, respectively. Increasing temperatures usually favor molecular mobility and intermolecular collisions, which accelerate the reaction rates up to an optimal temperature of catalysis, above which diminished reaction rates are observed due to enzyme denaturation. In the present study enzymatic measurements were not performed at temperatures higher than 40 °C in order to precisely determine the optimal temperature of catalysis, but in fact, the best observed rates for most tested substrates were at 40 °C.

**Figure 7:**
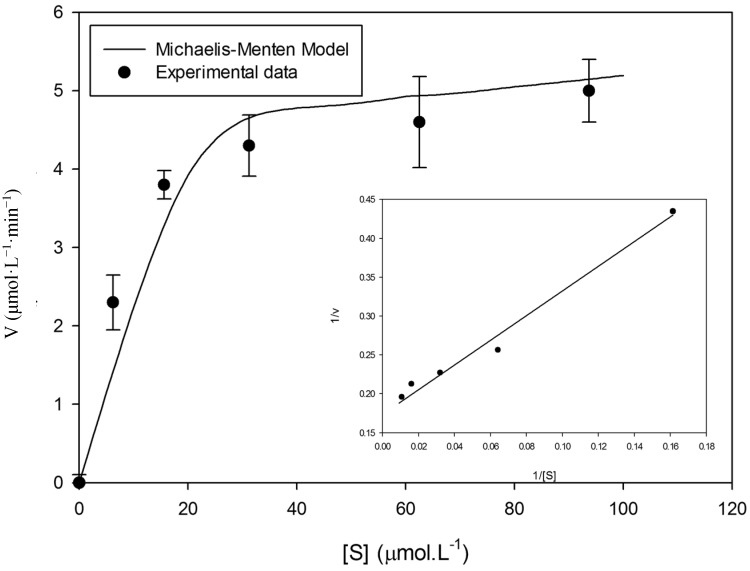
The influence of 2,6-DMP concentration on the initial reaction rates. Temperature of 40 °C and enzyme concentration of 4.8 U·mL^−1^. Each symbol (●) represents the average of three experimentally determined initial velocity measurements while error bars represent the respective standard deviation parameters. The line represents the fit of the data to Equation (2).

However, conversion rates using 3,4-xylenol, *p-*cresol and *o*-ethylphenol were higher at 30 °C as compared to 40 °C (Table 1). This observation indicated that optimal temperature of catalysis varies amongst the different substrates, likely due to divergences during the formation of enzyme-substrate complex and/or during catalysis. In fact, laccase optimal temperature of catalysis varies substantially among different species [36]. For example, the enzyme isolated from *Ganoderma lucidum* has an optimal temperature of catalysis between 20 and 25 °C, while optimal temperatures for the enzyme from *Marasmius quercophilus* lies between 40 and 50 °C [37]. The enzymatic activities are usually measured with syrigaldazine, ABTS, 2,6-DMP and guaiacol as substrate [38].

When 2,6-DMP was tested as substrate for immobilized laccase (Figure 7), we have observed hyperbolic behavior of the substrate saturation curve, confirming the enzyme follows Michaelis-Menten behavior.

### 2.3. Enzyme Reuse and Operational Stability Using Syringaldazine as Substrate

**Figure 8:**
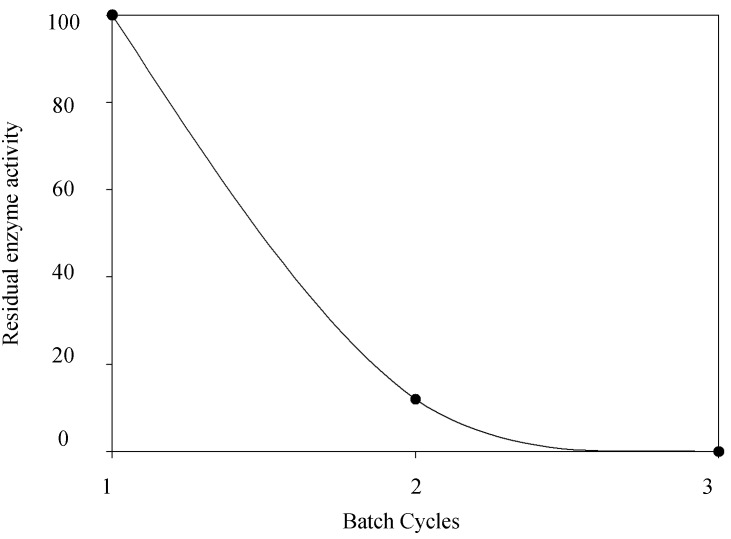
Residual enzyme activity after batch cycles of syringaldazine oxidation. Temperature of 40 °C, substrate concentration of 20 µmol·L^−1^ and enzyme concentration of 4.8 U·mL^−1^.

Regarding the recycling of laccase after a number of enzymatic cycles using syringaldazine as substrate, no satisfactory results were obtained (Figure 8).

In all studied conditions, there was a significant loss of enzymatic activity even after the second batch cycle. The most significant enzymatic activity loss was observed at 40 °C, and conversion rates between 10% and 20% of the initial catalytic activity after the second reaction cycle were observed. At lower temperatures (20 and 30 °C) activity after second cycle ranged between 30% and 60%, respectively. Even so, is still a condition that prevents the reuse of the enzyme and does not justify the immobilization strategy when syringaldazine is used as the substrate. Previous studies have investigated enzyme recycling strategies using immobilized laccase. Jiang and colleagues [39] used laccase immobilized on chitosan microspheres activated with glutaraldehyde for 2,2'-azino-bis3-ethylbenzothiazoline-6-sulphonic acid (ABTS) oxidation and obtained values after 10 batch cycles were 80% residual activity was maintained. D’Annibale and coworkers [14] immobilized laccase on glutaraldehyde activated chitosan beads and remarkably found that after 30 oxidative cycles the immobilized enzyme still presented 80% residual activity using ABTS as a substrate.

On the other hand, Rosa [40] investigated the use of Eupergit C immobilized laccase in the oxidation of effluents from the paper industry. In this study, enzymatic operational stability was investigated using syringaldazine as substrate. In accordance with our results, no satisfactory results were obtained, since most enzymatic activity had been lost after the second batch cycle. It is not clear why enzyme recycling is not feasible when syringaldazine is used as substrate.

In fact, two phenomena can be observed for laccase mediated syringaldazine oxidation. First, substrate inhibition occurs at high substrate concentrations leading to decreased rates of catalysis (Figure 5). Although substrate inhibition is frequently described in enzymology, our current knowledge does not allow excluding other possible molecular mechanisms for the observed inhibition. Second, immobilized laccase cannot be recycled and reused to oxidize syringaldazine, since enzyme inactivation also occurs (Figure 8). When the immobilized laccase is recycled and tested against 2,6-DMP, enzyme stability remains the same for several enzymatic batches, suggesting the immobilized enzyme is stable under tested conditions (Figure 1). However, under same experimental conditions, but using syringaldazine as substrate instead, the enzyme loses stability at the second batch cycle, suggesting that residual substrate or recently formed product might cause the observed enzyme inactivation. Therefore, when reaction is tested under initial velocity conditions (*i.e.*, <10% substrate consumption), inhibition is only observed at very high substrate concentrations (Figure 5). However, when enzyme operational stability is tested, reaction conditions do not follow initial velocity conditions and inactivating compounds may accumulate in greater amounts on the beads causing further inactivation during sequential batch cycles, as can be observed on Figure 8. Still, it is clear that further investigation is necessary to better understand the molecular mechanisms that lead to the enzymatic inactivation caused by syringaldazine oxidation and future experiments need to investigate the nature of observed inhibition.

Concerning 2,6-DMP as substrate, no substrate inhibition was observed, regardless of the utilized temperature (Figure 7) and the enzyme showed stability after eight batch cycles (Figure 1). The results corroborate the observations made by Rosa [40]. In their experiments the author conducted the oxidation of 2,6-DMP with laccase immobilized on Eupergit C. After completion of 10 reaction cycles, no significant reduction was observed in the activity of the immobilized enzyme, carrying out the process at room temperature and concentration 62.5 µmol·L^−1^.

## 3. Experimental Section

### 3.1. Chitosan Beads Production and Enzyme Immobilization

For chitosan beads production, chitosan powder (0.5 g) was dissolved in 5% (*w*/*v*) acetic acid (40 mL). The gel produced was added dropwise to a gently stirred 2 mol·L^−1^ NaOH aqueous solution and resultant beads were maintained in this solution during 12 h. Beads were washed with distilled water until the wash water reached a pH value of 8 and mixed with a 1% (*v*/*v*) aqueous solution of glutaraldehyde for crosslinking as previously reported [41]. Beads were further washed with distilled water for residual aldehyde removal until the wash water gave negative results for aldehyde detection using the Feder reagent [42]. Beads were then mixed to an enzyme solution containing 5.2 g·L^−1^ of laccase at pH 6 during 5 h.

### 3.2. Measurement of Laccase Enzymatic Activity Using Different Phenol Substituents

Different low molecular weight phenol derivatives were subjected to enzymatic bioconversion using chitosan immobilized laccase (Table 1). Assays were performed using 15 beads per assay (total enzymatic activity of 4.8 U·mL^−1^) and various substrates at 10 mg·L^−1^ in a 5 mL total reaction volume. 1 U is the amount of enzyme necessary to oxidize 1 µmol·L^−1^ of syringaldazine per minute (ε = 65.000 L·mol^−1^·cm^−1^). Reactions were maintained in water bath at 20, 30 or 40 °C during 24 h. Substrate consumption was estimated by measurement of phenolic compounds in the supernatant of the reaction mixture at time zero and 24 h later, according to the method described in Section 3.3. In order to investigate unspecific substrate adsorption into the beads we have mixed 15 cross-linked chitosan beads in the absence of enzyme and 10 mg·L^−1^ of phenolic compounds in 5 mL reaction mixture repetitive during 24 h. The percentage of phenol adsorption (%) onto the bead surface was estimated by measurement of the initial (C_initial_) and final (*C_final_*)supernatant phenol concentration and data was fitted to Equation (1):

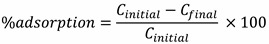
(1)

### 3.3. Measurement of Phenolic Compounds Concentration

Measurement of phenolic compounds concentration was performed using the 4-aminoantipyrine method as previously described [43]. The colorimetric detection of the dye formed upon oxidation of the phenol-containing sample in the presence of excess 4-aminoantipyrine allows easy phenol quantification. Briefly, after the enzymatic reaction was allowed to proceed for 24 h so that the phenolic compounds were oxidized, polymerized and precipitated, the insoluble products were easily removed from solution by filtering the reaction mixture through a 0.45 μm filter syringe. To this filtered sample (1 mL), were added ammonium buffer pH 10.0 (200 µL), sodium persulfate (5% *w*/*v*, 200 µL) and 4-aminoantipirine (2% *w*/*v*, 200 µL). Reaction was allowed to incubate during 10 min at room temperature and formation of produced dye was monitored spectrophotometrically at 504 nm. For 4-nitrophenol direct reading was performed at 405 nm.

### 3.4. Measurement of Kinetic Parameters Using Chitosan Immobilized Laccase

In order to evaluate kinetic parameters using chitosan immobilized laccase, two standard laccase substrates were used: syringaldazine and 2,6-dimethoxyphenol (2,6-DMP). Such compounds when oxidized produce colored products which are directly detected spectrophotometrically at 525 nm and 470 nm, respectively [35,44].

#### 3.4.1. Kinetic Parameters Using Syringaldazine as Substrate

Initial reaction rates (<10% substrate consumption) were measured using immobilized laccase and syringaldazine as substrate. Assays were performed in a 10 mL reaction volume containing tartarate buffer (pH 4.0), a fixed amount of immobilized enzyme and variable substrate concentrations (1.6; 3.2; 6.5; 13; 20; 25 and 38 µmol·L^−1^). Reaction mixtures were incubated with gentle stirring during 5 min and 1 mL sample was collected for spectrophotometric analysis at 525 nm. Assays were tested at different reaction temperatures (20, 30 or 40 °C). Reactions were performed at pH 4, since previous pH studies using immobilized laccase and syringaldazine as substrate have reported best enzymatic rates at this pH [45]. Individual substrate saturation data was fitted to Equation 2 using STATISTICA 7 Software.

#### 3.4.2. Kinetic Parameters Using 2,6-DMP as Substrate

Enzymatic reactions using 2,6-DMP as substrate were performed in a 10 mL reaction volume containing tartarate buffer (pH 5.0), a fixed amount of immobilized enzyme and variable substrate concentrations (6.2; 15.6; 31.2; 62.5 and 93.7 µmol·L^−1^). Reaction mixtures were incubated with gentle stirring during 5 min and 1 mL sample was collected for spectrophotometric analysis at 470 nm. Individual substrate saturation data was fitted to Equation (2) using STATISTICA 7 Software:

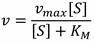
(2)
where:

*v* : reaction rate in µmol·L^−1^·s^−1^.

*v_max_* : maximum velocity in µmol·L^−1^·s^−1^.

*K_M_* : Michaelis Menten constant in µmol·L^−1^.

[𝑆] : substrate concentration in µmol·L^−1^.

### 3.5. Evaluation of Enzyme Recyclability

The recycling stability of the enzyme-chitosan beads was also examined. Therefore eight repeated enzymatic cycles were performed. Enzymatic reactions using syringaldazine or 2,6-DMP as substrate were performed in 10 mL reaction volume containing tartarate buffer pH 4.0 or 5.0, for syringaldazine and 2,6-DMP, respectively, a fixed amount of immobilized enzyme (30 U) and substrate concentrations of 20 µmol·L^−1^ (syringaldazine) and 93.7 µmol·L^−1^ (2,6-DMP). Reaction mixtures were incubated at 40 °C with gentle stirring during 5 min and 1 mL sample was collected for spectrophotometric analysis at 525 and 470 nm for syringaldazine and 2,6-DMP detection, respectively. Between each cycle, beads were carefully taken out from the reactor, washed with distilled water and re-placed in the next batch for the next enzymatic cycle. Efficiency of enzyme recycling was assessed by measurement of initial velocity at each cycle.

### 3.6. Evaluation of Enzyme Stability

Reactions containing syringaldazine as substrate, the immobilized-enzyme (50 beads, 250 U) or the equivalent amount of free enzyme were performed in 50 mL reaction volume containing phosphate buffer (pH 8.0). The mixtures were incubated at room temperature with gentle stirring and 1 mL sample was collected for enzyme assay (spectrophotometric analysis at 525 nm). Enzymatic measurements were performed once a day during a total of 30 days. Immobilized as well as free enzymes were stored at 8 °C.

## 4. Conclusions

In summary, chemical structure of the substrate is a key factor for the catalytic activity of immobilized laccase in the bioconversion of phenolic derivatives and substrate specificity does not change significantly upon laccase immobilization. After analysis of the enzyme stability after repeated cycles of reactions using syringaldazine as substrate, it is observed that the enzyme cannot be repeatedly recycled, and even after the second cycle the enzyme activity was only 40% of the initial activity. Thus 2,6-DMP is a better substrate to investigate laccase activity, since this substrate shows standard Michaelis Menten behavior and does not show substrate inhibition as opposed to syringaldazine. The immobilization strategy presented here allowed the use of multiple cycles of enzymatic reaction (using 2,6-DMP) without substantial loss of enzymatic activity, allowing its use in industrial processes, especially in the bioconversion of phenolic derivatives in industrial waste water. Additionally, immobilization resulted in a significant increase in enzymatic stability when tested for a period of 30 days.
